# Beyond the Gut: Brain Fog, Sleep Quality, Cognitive Function and Quality of Life in Celiac Disease

**DOI:** 10.3390/nu18142365

**Published:** 2026-07-19

**Authors:** Canan Altinsoy, Evrim Kahramanoğlu Aksoy, Mehmet Raşit Ayte, Derya Dikmen

**Affiliations:** 1 Department of Nutrition and Dietetics, Faculty of Health Sciences, Recep Tayyip Erdoğan University, 53350 Rize, Türkiye; canan.altinsoy@erdogan.edu.tr; 2 Department of Nutrition and Dietetics, Faculty of Health Sciences, Hacettepe University, 06230 Ankara, Türkiye; 3 Department of Gastroenterology, Ankara Atatürk Sanatoryum Training and Research Hospital, University of Health Sciences, 06290 Ankara, Türkiye

**Keywords:** celiac disease, brain fog, cognitive functions, sleep quality, Montreal cognitive assessment, MoCA, gut–brain axis

## Abstract

**Background/Objectives**: This exploratory comparative cross-sectional observational study investigated brain fog symptoms, cognitive function, sleep quality, quality of life, and selected serum biomarkers related to inflammation and neurocognitive function in newly diagnosed patients with celiac disease (ND-CeD), patients with CeD on a gluten-free diet (GFD-CeD), and controls. **Methods**: A total of 62 participants were included: ND-CeD patients (*n* = 18), GFD-CeD patients (*n* = 17), and healthy controls (*n* = 27) with no statistically significant differences in age or sex distribution across groups. Brain fog symptoms and severity, cognitive function, sleep quality, and quality of life were assessed using the Brain Fog Scale (BFS), Brain Fog Severity Score (BFSS), Montreal Cognitive Assessment (MoCA), Single-Item Sleep Quality Scale (SQS), and World Health Organization Quality of Life Questionnaire-Brief Form-TR (WHOQOL-BREF-TR), respectively. Serum BDNF, S100B, TLR4, IL-6, and nitric oxide (NO) levels were measured by ELISA. **Results**: ND-CeD patients had higher BFSs and BFSSs and lower MoCA, SQS, and WHOQOL-BREF-TR scores than healthy controls (*p* < 0.05). GFD-CeD patients showed numerically intermediate or more favorable scores than ND-CeD patients in several outcomes; however, most differences from controls were not statistically significant. Compared with ND-CeD patients, GFD-CeD patients had higher WHOQOL-BREF-TR General Health, Psychological Health, and Social Relationships scores (*p* < 0.05). In exploratory within-group analyses, after correction for multiple comparisons, higher BFS scores were associated with poorer psychological health and lower MoCA scores, and higher MoCA scores were associated with better psychological and physical health domains, particularly in the ND-CeD group. In the adjusted regression model, older age, income status, and newly diagnosed disease status were independently associated with MoCA scores. No statistically detectable between-group differences were observed in serum IL-6, NO, BDNF, S100B, or TLR4 levels. **Conclusions**: These preliminary findings suggest that brain fog symptoms, cognitive performance, sleep quality, and quality of life may deserve greater attention at diagnosis and during follow-up in celiac disease. Although GFD-CeD patients showed more favorable scores in some outcomes, these cross-sectional differences should not be interpreted as treatment-related improvement. Larger longitudinal studies with objective assessment of gluten-free diet adherence, disease activity, micronutrient status, sleep quality, and gut–brain axis-related biomarkers are needed to confirm these findings.

## 1. Introduction

Celiac disease (CeD) is an autoimmune enteropathy triggered by the ingestion of gluten-containing food in genetically susceptible individuals and is characterized by specific serological markers and, when present, histological evidence of small-intestinal enteropathy [1,2]. Epidemiological studies estimate that celiac disease affects around 1% of the population, yet most cases remain undiagnosed, reflecting its broad clinical variability [3]. This diagnostic gap is well illustrated by the “celiac iceberg” model, where symptomatic cases are just the visible part, while most, consisting of silent, atypical, and potential cases, lie beneath the surface [3,4,5]. Although it was historically considered a condition limited to the gastrointestinal (GI) tract with classic symptoms such as diarrhea, abdominal pain, weight loss, and malabsorption, current understanding indicates that the autoimmune response in celiac disease is not confined to the intestine but can exert systemic effects [6,7]. Accumulating evidence indicates that CeD can manifest with a wide range of extraintestinal features involving the musculoskeletal, neurological, endocrine, dermatological, and hepatic systems, highlighting the multisystemic nature of the disease [8]. Within this broad systemic profile, increasing attention is being drawn to the neurological and neurocognitive manifestations of the disease [9]. In particular, central nervous system–related symptoms and patient-reported cognitive complaints have become increasingly prominent in recent discussions [10,11,12,13,14,15]. Reported neurological complications include cerebellar ataxia, peripheral neuropathy, seizures, headaches, cognitive impairment, and various neuropsychiatric symptoms [16,17,18]. Beyond these well-defined disorders, individuals with CeD frequently describe more subtle cognitive and behavioral difficulties [19,20,21], often referred to as “brain fog”, such as reduced concentration, forgetfulness, mental slowness, word-finding problems, confusion, and impairments in attention, memory, and executive functioning, which may substantially affect daily functioning and quality of life [11,22,23].

Beyond cognitive complaints, sleep disturbances are also increasingly reported in individuals with CeD and may further contribute to difficulties in attention, memory, and daytime functioning. Sleep complaints (e.g., poor sleep quality, increased sleep disturbance, and insomnia) are reported more frequently in celiac disease, but findings on the impact of a GFD on sleep outcomes remain inconsistent [24,25,26,27]. These difficulties are thought to stem from GI symptoms, immune-inflammatory mechanisms, altered gut–brain axis (GBA) signaling, and disease-related micronutrient deficiencies [28]. In line with this conceptual framework, we previously reported that brain fog symptoms were positively associated with GI symptoms and inversely associated with sleep quality [29]. Still, the extent to which these cognitive and neuropsychiatric manifestations reflect the psychosocial burden of living with a chronic disease or represent disease-specific pathophysiological processes remains unclear [30,31].

Comparing newly diagnosed untreated CeD patients with GFD-treated CeD patients and healthy controls may help clarify whether brain fog symptoms, cognitive performance, sleep quality, and quality of life differ across clinically relevant disease states. Newly diagnosed patients represent the untreated or active diagnostic phase, in which gastrointestinal symptoms, immune-inflammatory activity, malabsorption-related factors, and micronutrient deficiencies may be more pronounced [16,32,33]. In contrast, GFD-treated patients represent a clinically followed diet-treated group in whom some disease-related features may be less pronounced, although residual symptoms and diet-related psychosocial burden may persist [34,35]. Importantly, to our knowledge, evidence directly comparing untreated and GFD-treated adult CeD patients with healthy controls in terms of brain fog symptoms, cognitive per-formance, sleep quality, and quality of life remains limited [23,36]. Therefore, including both CeD groups and healthy controls provides a framework for examining whether neurocognitive and sleep-related outcomes are more prominent around diagnosis, persist during follow-up, or show values closer to those of controls. However, because such comparisons are cross-sectional, they cannot be interpreted as evidence of within-person improvement after a GFD.

The gut–brain axis (GBA) is a bidirectional network linking the central and enteric nervous systems, facilitating communication between the brain’s emotional and cognitive centers and GI functions. It integrates neural, immune, and endocrine pathways to maintain gut balance and influence emotion, motivation, and cognition. The GBA coordinates responses via immune activation, intestinal permeability, and enterondocrine signaling [37]. Given the central involvement of immune and inflammatory signaling within the GBA, defects in these pathways are particularly relevant in CeD, where gluten-induced intestinal inflammation, increased epithelial permeability, and systemic immune activation can lead to changes in cytokine profiles and neuroimmune communication [38]. Recent studies indicate that celiac disease is associated with elevated levels of several inflammatory mediators, which may not only reflect intestinal immune activation but also influence gut–brain signaling and cognitive outcomes [39,40]. These immune–inflammatory alterations provide a biologically plausible pathway linking celiac disease with neurological and neurocognitive manifestations, supporting the relevance of assessing systemic inflammatory and innate immune activity. Mechanistic evidence in celiac disease has predominantly come from mucosal biopsy–based studies, and links between local intestinal immune activation and systemic (serum) inflammation remain insufficiently characterized. Most cytokine studies have relied on duodenal/jejunal biopsies and local assessment by immunohistochemistry or in situ hybridization, whereas comparatively few have measured serum cytokines using ELISA [41]. Accordingly, this study aimed to assess circulating IL-6, NO-related markers, and TLR4. Beyond inflammatory and innate immune pathways, neurocognitive complaints may also relate to altered neurotrophic signaling and glial responses [42,43]. Brain-derived neurotrophic factor (BDNF) is a neurotrophin involved in synaptic plasticity and cognitive function [43,44], whereas S100B is an astrocyte-derived calcium-binding protein that has been used as a circulating marker linked to neuroglial activity and blood–brain barrier perturbation [45,46]. Therefore, we also examine serum BDNF and S100B as exploratory biomarkers. Collectively, the available evidence suggests that CeD may be linked to cognitive and sleep-related complaints and reduced quality of life, potentially through gut–brain axis pathways and the broader clinical symptom burden of the disease [16,24,27,33]. Therefore, this study aimed to compare brain fog symptoms, cognitive performance, sleep quality, and quality of life among newly diagnosed CeD patients (ND-CeD), CeD patients following a gluten-free diet (GFD-CeD), and healthy controls, and to explore between-group differences in circulating neuroinflammatory and neuroglial markers, including BDNF, S100B, IL-6, TLR4, and NO.

We hypothesized that ND-CeD patients would show a higher burden of brain fog symptoms, poorer sleep quality, lower cognitive performance, and lower quality-of-life scores compared with healthy controls. We also hypothesized that GFD-CeD patients would show more favorable scores than ND-CeD patients in some patient-reported and cognitive outcomes, while recognizing that the cross-sectional design does not allow causal or longitudinal interpretation. In addition, we expected that brain fog symptoms, sleep quality, cognitive performance, and quality of life would be interrelated, and that serum biomarkers would provide exploratory information regarding potential gut–brain axis-related differences across groups.

## 2. Materials and Methods

This comparative cross-sectional observational study included three groups: newly diagnosed celiac disease patients (ND-CeD), gluten-free diet-treated celiac disease patients (GFD-CeD), and healthy controls. The research was conducted at Ankara Atatürk Sanatory Education and Research Hospital between 1 August 2024 and 1 July 2025.

### 2.1. Participants

G*Power 3.1.9.7 was used for sample size and power considerations. The initial sample size calculation was based on serum BDNF levels reported by Russo et al. in patients with celiac disease [47]. However, because the published BDNF values were reported as means ± SEM rather than means ± SD and were derived from a within-subject comparison in a longitudinal design, they were not considered an appropriate direct basis for the present independent three-group, between-subjects design. To provide a more transparent assessment of statistical power, a sensitivity power analysis was additionally performed based on the achieved sample size. With 62 participants across three groups, α = 0.05, and 80% power, the study was powered to detect Cohen’s f = 0.404 for one-way ANOVA comparisons. Accordingly, serum biomarker analyses were considered exploratory, and smaller biomarker effects may not have been detected.

Participants were classified into three distinct groups. The ND-CeD group included patients who had recently been diagnosed with celiac disease based on celiac disease-specific serological tests and small-intestinal biopsy and who had not yet started a gluten-free diet. The GFD-CeD group included patients with a previous diagnosis of celiac disease who had followed a gluten-free diet for at least two years. This threshold was selected to represent longer-term dietary treatment, as mucosal recovery in adults with celiac disease may require prolonged gluten-free diet adherence and has been evaluated at two years and beyond in previous follow-up studies [48,49]. The control group included healthy individuals without known medical conditions. Celiac disease-related serological markers were assessed during the study period in the GFD-CeD and control groups. These markers were negative in the GFD-CeD group, supporting serological remission, and negative in the control group, helping to exclude unrecognized celiac disease. Participants were directed to the appropriate study group by the responsible gastroenterologist based on clinical assessment and serological findings. In addition, GFD-CeD patients were asked to report their gluten-free diet duration and self-reported adherence. However, objective dietary assessment tools (e.g., gluten immunogenic peptides in urine or stool) and histological confirmation of mucosal healing were not performed, which is acknowledged as a limitation of the present study [50,51]. Healthy controls were recruited during the same study period using a convenience-based community sampling approach through social media and messaging application postings, community announcements, and word-of-mouth referrals. During recruitment, efforts were made to select controls with a similar age and sex distribution to the CeD groups. Eligible controls were screened to confirm the absence of known medical conditions and negative celiac disease-related serology. All participants were invited for a single study visit. During the same visit, face-to-face interviews, questionnaire assessments, cognitive assessment, and blood sampling were performed. Face-to-face interviews, questionnaire assessments, and cognitive assessment were conducted by the first author, according to a standardized study protocol. Blood samples were collected by the responsible clinical nurse as part of routine hospital procedures during the same study visit.

Exclusion criteria were: a history of malignancy or chemotherapy; pregnancy or breastfeeding; post-menopausal status; acute or chronic inflammatory disease or infection; strenuous physical activity; severe psychiatric disorders; substance abuse; antidepressant use; surgery within the past month; neurological disease (e.g., brain tumor, stroke); use of prebiotics/probiotics or dietary supplements; prior intensive care unit treatment for COVID-19; shift work; adherence to a special diet program; chronic medications affecting sleep patterns; other primary GI disease; migraine; self-reported GI infection within 2 weeks prior to enrollment; antibiotic use within 12 weeks prior to enrollment; and unexplained rectal bleeding.

### 2.2. Data Collection

Data were collected via face-to-face interviews using a personal information form and the Brain Fog Scale (BFS), Brain Fog Severity Score (BFSS), Single-Item Sleep Quality Scale (SQS), Montreal Cognitive Assessment (MoCA), and World Health Organization Quality of Life Questionnaire-Short Form (WHOQOL-BREF-TR).

Personal Information Form: A researcher-developed form consisting of questions designed to determine participants’ socio-demographic characteristics.

Brain Fog Scale (BFS): The BFS is a 23-item self-report scale developed to assess brain fog symptoms in clinical and research settings [52]. The Turkish validity and reliability study was conducted by Baş et al. [53]. Items are rated on a 5-point Likert scale ranging from 1 (“never”) to 5 (“always”), with higher scores indicating greater brain fog symptom burden. The scale has no reverse-coded items and no established cutoff point. It includes three subscales: confusion, mental fatigue, and impaired cognitive acuity. A sample item is “Konsantre olmakta zorlandım” (“I had difficulty concentrating”). The Turkish version has demonstrated good psychometric properties [53].

Brain Fog Severity Score (BFSS): The BFSS assesses perceived brain fog severity using a single visual numeric scale ranging from 0 to 100 in 10-point increments. Higher scores indicate greater brain fog severity [54]. Participants were asked to rate the overall severity of their brain fog [54].

Single-Item Sleep Quality Scale (SQS): Sleep quality was assessed using the SQS (SQS), a self-rated global measure of perceived sleep quality over the previous seven days. Participants were asked to rate their overall sleep quality on an 11-point scale ranging from 0 to 10, where 0 indicates “terrible” and 10 indicates “excellent.” While responding, participants were instructed to consider sleep duration, ease of falling asleep, nocturnal awakenings, early morning awakenings, and how refreshing their sleep was. Scores were categorized as terrible (0), poor (1–3), fair (4–6), good (7–9), and excellent (10). In the present study, the SQS was used as a brief global screening measure rather than as a comprehensive multidimensional sleep assessment [55].

The Montreal Cognitive Assessment (MoCA): Cognitive performance was assessed using the MoCA, a brief screening tool developed to detect mild cognitive impairment [56]. The Turkish validity study was conducted by Selekler et al. [57]. The MoCA evaluates multiple cognitive domains, including attention, concentration, executive function, memory, language, visuospatial skills, abstraction, calculation, and orientation. Total scores range from 0 to 30, with higher scores indicating better cognitive performance. In accordance with standard MoCA administration guidelines, one point was added to the total score for participants with 12 or fewer years of formal education, as recommended in the original validation study [56]. In accordance with the Turkish validation study, scores of 20 or below were considered indicative of possible cognitive impairment [57]. The MoCA was administered through the official MoCA program by the first author, who was trained and certified for MoCA administration.

The World Health Organization Quality of Life (WHOQOL-BREF-TR): Quality of life was assessed using the WHOQOL-BREF-TR, the Turkish version of the WHOQOL-BREF developed by the World Health Organization to evaluate perceived quality of life [58,59]. The Turkish validity and reliability study was conducted by Eser et al. (1999) [59]. The WHOQOL-BREF-TR consists of 27 items, including one national item added during the Turkish adaptation, and assesses four domains: physical health, psychological health, social relationships, and environment. Scores can be transformed to either a 0–20 or 0–100 scale, with higher scores indicating better quality of life. In this study, domain scores were transformed to a 0–100 scale. The Turkish version has shown acceptable internal consistency, with Cronbach’s alpha values of 0.83, 0.66, 0.53, and 0.73 for the physical, psychological, social relationships, and environment domains, respectively [59].

Serum collection and laboratory measurements: For all participants, a single blood sample was taken in the early morning after a minimum 10–12 h fast to measure BDNF, S100B, IL-6, TLR4, and NO levels in serum. Serum was separated and stored at −80 °C until analysis. Serum BDNF, S100B, IL-6, TLR4, and NO concentrations were measured using ELISA kits (BT LAB, Shanghai, China) according to the manufacturer’s instructions.

### 2.3. Statistical Analysis

Statistical analyses were performed using IBM SPSS Statistics for Windows, version 26.0 (IBM Corp., Armonk, NY, USA). Normality was assessed using the Shapiro–Wilk test prior to analysis. Continuous variables are presented as mean ± standard deviation (SD) for normally distributed data or median (interquartile range, IQR) for non-normally distributed data, and categorical variables as n (%). Group comparisons were conducted using one-way ANOVA (with Bonferroni post hoc tests when variances were homogeneous and Tamhane’s T2 post hoc tests when variances were not homogeneous) or the Kruskal–Wallis H test (with Bonferroni-adjusted pairwise comparisons). For variables showing deviations from normality in one or more groups (BFSS and SQS), Kruskal–Wallis tests were additionally conducted and yielded results consistent with the ANOVA findings, supporting the robustness of the reported results. Categorical variables were compared using the chi-square test or Fisher’s exact test when expected cell counts were <5. Associations between continuous variables were assessed using Pearson’s or Spearman’s correlation, as appropriate, and are reported as r with corresponding *p* values. For within-group correlation analyses, Benjamini–Hochberg false discovery rate (FDR) correction was applied separately within each group to address multiple comparisons; FDR-adjusted q values are reported alongside uncorrected *p* values. Two-step hierarchical multivariable linear regression models were built to identify independent associations. Given the exploratory nature of the study and the modest sample size relative to the number of predictors, regression findings should be interpreted as hypothesis-generating, and adjusted R^2^ is reported as the primary measure of model fit. A two-sided *p*-value < 0.05 was considered statistically significant.

## 3. Results

### 3.1. Participant Characteristics

A total of 62 participants were included in the study: 18 newly diagnosed untreated celiac disease patients, 17 celiac disease patients following a gluten-free diet, and 27 controls. As shown in Table 1, age, sex, and education level did not differ significantly among the groups (*p* > 0.05). Although sex distribution did not differ significantly, the GFD-CeD group had a higher proportion of women than the other groups. Significant differences were observed in income level, smoking status, and regular physical activity (*p* < 0.001, *p* < 0.001, and *p* = 0.001, respectively). Controls had a higher proportion of individuals with income above the national minimum wage and regular physical activity, whereas smoking was observed only among celiac disease patients.

### 3.2. Sleep Quality and Cognitive Status According to Study Groups

The distribution of participants’ sleep quality and MoCA-defined cognitive status by study group is presented in Table 2. Sleep quality differed significantly among the groups (*p* = 0.015). Poorer sleep quality was more common in the ND-CeD group, good sleep quality was most prevalent in the control group, and the GFD-CeD group showed an intermediate distribution. Similarly, MoCA-defined cognitive status differed significantly among the groups (*p* < 0.001). The proportion of participants with possible cognitive impairment was highest in the ND-CeD group, lower in the GFD-CeD group, and lowest in the control group.

### 3.3. Brain Fog Symptoms, Cognitive Performance, Sleep Quality, and Quality of Life Across Groups

Brain fog symptoms, cognitive performance, sleep quality, and quality-of-life scores were compared across the three study groups, as shown in Table 3. Significant group differences were observed in total BFS score, impaired cognitive acuity, BFSS, MoCA score, SQS score, and all WHOQOL-BREF-TR domains. Overall, ND-CeD patients showed the least favorable profile, with higher BFSs and BFSSs and lower MoCA, SQS, and quality-of-life scores compared with controls. GFD-CeD patients generally showed intermediate or closer-to-control values for several outcomes. Post hoc analyses indicated that ND-CeD patients differed significantly from controls for total BFS, impaired cognitive acuity, BFSS, MoCA, SQS, and selected WHOQOL-BREF-TR scores. In addition, GFD-CeD patients had significantly higher psychological health, and social relationship scores than ND-CeD patients.

In exploratory adjusted analyses controlling for age, income status, and regular physical activity, the ND-CeD group had significantly lower adjusted sleep quality scores than controls (B = −2.00, 95% CI: −3.66 to −0.34, *p* = 0.019), whereas no significant difference was observed between the GFD-CeD and control groups (Supplementary Table S2). For brain fog symptoms, the unadjusted difference between the ND-CeD and control groups was attenuated after adjustment; neither the ND-CeD versus control contrast nor the GFD-CeD versus control contrast remained statistically significant in the fully adjusted model (Supplementary Table S3).

For WHOQOL-BREF-TR outcomes, adjustment did not materially alter the overall pattern of group differences. Compared with controls, the ND-CeD group had significantly lower adjusted scores in the physical health (B = −7.33, 95% CI: −11.21 to −3.45, *p* < 0.001), psychological health (B = −3.98, 95% CI: −6.87 to −1.10, *p* = 0.008), social relationships (B = −2.60, 95% CI: −4.36 to −0.84, *p* = 0.004), and environment domains (B = −5.21, 95% CI: −9.14 to −1.27, *p* = 0.010). The GFD-CeD group also had lower adjusted physical health scores than controls (B = −3.67, 95% CI: −7.09 to −0.26, *p* = 0.036), whereas no other adjusted differences between the GFD-CeD and control groups were statistically significant (Supplementary Table S4).

### 3.4. Correlations Between Brain Fog, Cognitive Performance, Sleep Quality, and Quality of Life

**Figure 1:**
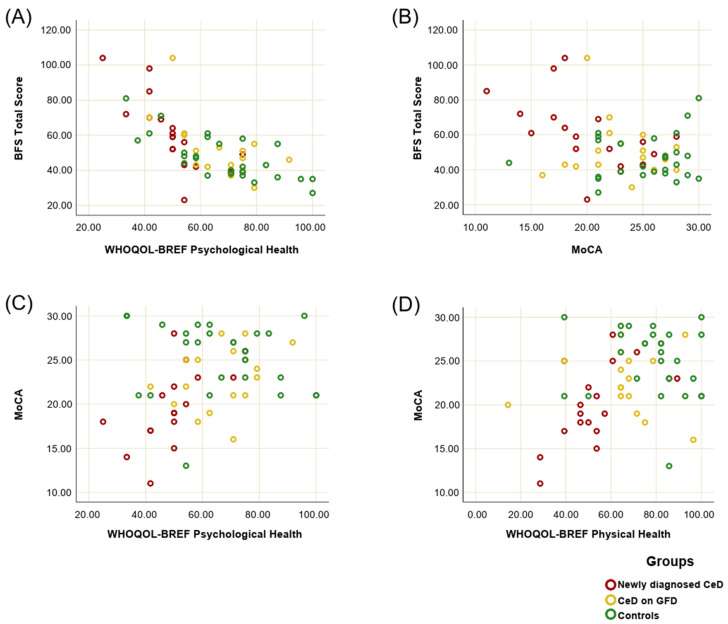
**Scatter plots of associations between brain fog symptoms, cognitive performance, and quality of life.** Scatter plots illustrate the four associations that remained statistically significant after Benjamini–Hochberg false discovery rate (FDR) correction in the ND-CeD group (q < 0.05; see Table 4): (**A**) BFS total score and WHOQOL-BREF-TR Psychological Health, (**B**) BFS total score and MoCA score, (**C**) MoCA score and WHOQOL-BREF-TR Psychological Health, and (**D**) MoCA score and WHOQOL-BREF-TR Physical Health. For visual comparison, data points from all three study groups are displayed; however, the statistically significant associations highlighted in this figure were identified in the ND-CeD group. BFS, Brain Fog Scale; MoCA, Montreal Cognitive Assessment; WHOQOL-BREF-TR, World Health Organization Quality of Life Questionnaire-Brief Form-Turkish.

In the ND-CeD group, following Benjamini–Hochberg false discovery rate (FDR) adjustment across 13 correlations, BFS scores were negatively correlated with WHOQOL-BREF-TR Psychological Health (r = −0.917, q = 0.004) and MoCA scores (r = −0.693, q = 0.004). MoCA scores were positively correlated with WHOQOL-BREF-TR Psychological Health (r = 0.750, q = 0.004) and Physical Health (r = 0.628, q = 0.016). The four associations that remained statistically significant after FDR adjustment are illustrated in Figure 1. No correlations remained statistically significant after FDR adjustment in the GFD-CeD group. Unadjusted *p* values and FDR-adjusted q values for all tested associations are presented in Table 4.

### 3.5. Serum Biomarkers Across Study Groups

In the analysis of serum parameters, no statistically detectable between-group differences were observed in serum BDNF, S100B, TLR4, IL-6, or NO levels (*p* > 0.05) (Table 5). These findings should be interpreted in the context of the limited statistical power of the present study for biomarker comparisons (sensitivity analysis: Cohen’s f = 0.404); smaller or clinically meaningful differences cannot be excluded. Kruskal–Wallis effect sizes were small (η^2^H range: 0.013–0.031), suggesting limited between-group separation for these biomarkers in the present sample. Kruskal–Wallis η^2^H values are presented in Table 5, and pairwise Hedges’ g values with 95% confidence intervals are provided in Supplementary Table S1. Boxplots of serum biomarker levels across groups are presented in Supplementary Figures S1 and S2.

### 3.6. Factors Associated with MoCA Total Score

Hierarchical multiple linear regression was conducted to identify factors associated with MoCA total scores (Table 6). Model 1 included age, income status, and regular physical activity and explained 32.5% of the variance in MoCA scores (adjusted R^2^ = 0.278, *p* < 0.001). Older age was associated with lower MoCA scores, whereas income above the national minimum wage was associated with higher MoCA scores.

In Model 2, group status was additionally entered. Although the increase in explained variance did not reach the conventional level of statistical significance (ΔR^2^ = 0.064, *p* for F change = 0.066), the adjusted ND-CeD versus control contrast was statistically significant, with newly diagnosed patients having lower MoCA scores than controls (B = −3.46, 95% CI: −6.37 to −0.56, *p* = 0.020). No statistically significant difference was observed between the GFD-CeD and control groups. The fully adjusted model explained 38.9% of the variance in MoCA scores (adjusted R^2^ = 0.322).

Descriptive micronutrient data across study groups are presented in Supplementary Table S5. To assess whether micronutrient status could account for the observed association, a sensitivity analysis additionally including ferritin, folate, and vitamin B12 as covariates was conducted. The adjusted ND-CeD versus control contrast remained statistically significant, with newly diagnosed patients having lower MoCA scores than controls (B = −3.42, 95% CI: −6.59 to −0.25, *p* = 0.035) (Supplementary Table S6).

## 4. Discussion

In this exploratory cross-sectional study, brain fog symptoms, cognitive performance, sleep quality, and quality of life were assessed across three clinically defined groups: newly diagnosed celiac disease patients (ND-CeD), celiac disease patients following a gluten-free diet (GFD-CeD), and healthy controls. The groups did not differ significantly in age or sex distribution. The main findings were that ND-CeD patients showed a higher brain fog symptom burden, lower cognitive performance, poorer sleep quality, and lower quality-of-life scores than healthy controls, whereas GFD-CeD patients showed intermediate or more favorable scores for several outcomes, with no statistically significant differences from controls in most comparisons. No between-group differences were detected in serum biomarker levels, a finding that should be interpreted in the context of the limited statistical power of the present study for biomarker comparisons. Examining these domains concurrently across clinically relevant disease states provides a more comprehensive picture of the extraintestinal burden of celiac disease and may contribute to the limited evidence comparing untreated and diet-treated adult patients with healthy controls in these areas.

In the present study, ND-CeD patients exhibited significantly higher brain fog symptom scores and greater brain fog symptom severity than healthy controls. Importantly, these differences were not observed in patients adhering to a GFD, although this group still tended to report higher brain fog symptoms and severity than controls. These cross-sectional findings indicate that brain fog symptom burden and severity were higher in the untreated/active celiac disease group than in the treated celiac disease group, with comparisons against controls showing a similar pattern. Lebwohl and Ludvigsson (2014) noted that clinicians frequently hear patients describe “brain fog,” sometimes following accidental gluten exposure and sometimes as a presenting symptom that can be slow to resolve after adopting a GFD [60]. Research findings remain limited, but some longitudinal observations suggest that cognitive changes may track, at least in part, with disease activity and treatment response [16,33,36]. For example, in a pilot longitudinal study, Lichtwark and colleagues reported that selected measures of objectively assessed cognitive processing speed were moderately associated with time on a GFD, serologic activity, and histologic activity in the duodenal mucosa; while not definitive, these observations are broadly consistent with the possibility that intestinal inflammation and treatment status may relate to cognitive complaints in some patients [36]. More broadly, the clinical relevance of brain fog in celiac disease is increasingly recognized, and available literature suggests that these symptoms are common, frequently described in terms of attention, memory, and mental clarity, and may be measurable in at least some contexts; several reports also note potential improvement with strict gluten withdrawal, although mechanisms remain incompletely understood and such observations derive from longitudinal studies and cannot be inferred from the present cross-sectional design [18,23,61,62,63]. Although conducted in a neurological gluten-related disease cohort rather than a classical CeD cohort, Croall et al. also reported brain fog as an acute symptom after gluten exposure in 28.6% of respondents, further supporting the relevance of subjective cognitive complaints across the gluten-related disorder spectrum [15]. A nationwide online study conducted by Edwards George et al. (2022) [13] indicated that brain fog after presumed gluten exposure is often described by patients as difficulty concentrating, forgetfulness and grogginess. This study highlighted that brain fog after presumed gluten exposure was common, affecting 89% of a cohort of 1143 celiac patients [13]. Taken together, these studies underscore that brain fog is a common and clinically meaningful complaint in celiac disease; however, mechanistic understanding is still limited. Accordingly, the literature has put forward several hypotheses, most plausibly involving gut–brain axis–related pathways, while acknowledging that causal pathways remain to be clarified [64,65,66]. One possible explanation for this pattern is the greater GI symptom burden often present around diagnosis, which may contribute to poorer sleep continuity and daytime cognitive clouding [67]. Consistent with this interpretation, our previous work showed that greater GI symptom burden was associated with more brain fog symptoms and that higher brain fog severity was related to poorer sleep quality [29]. Consistent with a diagnosis-phase symptom burden framework, untreated celiac patients have also been reported to exhibit higher GI symptom scores than those adhering to a GFD [68]. Additionally, brain fog-like symptoms at diagnosis may have multiple causes, with micronutrient deficiencies, especially iron deficiency and iron-deficiency anemia, potentially playing a role in fatigue and impaired concentration, which can increase subjective cognitive complaints [69]. In our study, brain fog symptoms were also meaningfully related to quality of life: among ND-CeD patients, brain fog symptoms were significantly associated with WHOQOL-BREF-TR Physical Health and WHOQOL-BREF-TR Psychological Health, and among GFD-treated patients, significant associations were observed with WHOQOL-BREF-TR Psychological Health. This pattern may suggest that physical quality-of-life domains differ between disease states, whereas the perceived cognitive and mental burden may remain notable regardless of treatment status; however, these are cross-sectional observations across different individuals and should not be interpreted as evidence of within-person change. Sample size and differences in residual symptoms may also have contributed to this pattern [15]. As the functional burden of brain fog becomes clearer, it may require more systematic clinical attention in CeD [70,71]. Notably, despite its negative impact, the underlying mechanisms and clinical implications of brain fog remain insufficiently studied [61,72]. Overall, our findings extend the limited evidence by linking brain fog to patient-reported functional outcomes, but well-designed longitudinal studies controlling for key confounders (e.g., sleep disturbance and mood symptoms) are needed to clarify mechanisms and clinical significance [72].

In our study, participants’ cognitive performance was assessed using the MoCA, and ND-CeD patients’ MoCA scores were significantly lower than those of the control group. Importantly, in the fully adjusted model, the cognitive disadvantage was specific to the ND-CeD group, as ND-CeD patients had significantly lower MoCA scores than healthy controls, whereas no significant difference was observed between GFD-treated patients and controls (Table 6). MoCA is a widely used screening tool for mild cognitive impairment and has been used in numerous studies to assess cognitive function in patients with CeD [18,73,74,75,76]. Our findings are consistent with studies showing that ND-CeD patients, even if neurologically asymptomatic, have significantly lower MoCA scores compared to healthy control groups [73,74,77]. The frequency of neurological findings in CeD varies depending on the studies and sample characteristics; however, it is noted that a significant proportion of neurological complaints are reported at the onset of the disease [23,33,36,78,79]. Hu et al.’s case series further supports the plausibility of cognitive involvement in celiac disease by describing 13 adults who developed progressive cognitive decline temporally linked to symptomatic onset or exacerbation of biopsy-proven celiac disease. Patients frequently exhibited a fronto-subcortical pattern of impairment, and neurological comorbidity was common (ataxia and, in some cases, peripheral neuropathy), while neuroimaging and EEG findings were largely non-specific [78]. Supporting this, in a 3-year prospective cohort of 100 consecutive adults ND-CeD in a secondary-care gastroenterology clinic, Hadjivassiliou et al. found neurological symptoms/signs to be common (e.g., headaches and gait/coordination disturbances), with abnormal brain imaging in 60% of patients, including abnormal cerebellar MR spectroscopy in 47% and white matter lesions beyond those expected for age in 25% [16]. In their 7-year follow-up of a subset of this cohort, headache prevalence decreased after initiation of a GFD, whereas the prevalence of incoordination increased, partly due to new-onset cases observed only among patients who remained seropositive for one or more gluten-related antibodies. Persistent seropositivity was also associated with a higher rate of cerebellar grey matter atrophy on repeat neuroimaging [33]. Population-based data also support this picture [12]. In their UK Biobank–based analysis, Croall et al. (2020) [12] matched 104 participants with celiac disease to healthy controls on age, sex, education level, body mass index, and hypertension status. Compared with controls, individuals with celiac disease demonstrated significantly slower reaction time. Tract-based spatial statistics (TBSS; a whole-brain white-matter diffusion analysis) identified widespread increases in axial diffusivity (a diffusion MRI metric often interpreted as altered axonal microstructure), whereas voxel-based morphometry (a measure of regional grey-matter volume) and Fazekas ratings (a clinical scale for white-matter hyperintensities) did not differ between groups. The authors concluded that celiac disease in this population sample was associated with a cognitive deficit (slower reaction time) and white matter changes based on brain imaging analyses [12]. These impairments typically affect memory, attention, and processing speed, and may correlate with disease activity and cerebral changes seen on imaging [12,80]. Taken together, available evidence indicates that cognitive impairment in CeD may relate to disease activity and, in some cases, neuroimaging findings such as white matter alterations or altered cerebral hemodynamics [12,74,80]. In our cross-sectional analysis, the GFD-followed group had MoCA scores comparable to those of controls. Although we did not follow the same individuals over time, the finding that MoCA scores in the GFD-CeD group were comparable to those of controls represents a cross-sectional observation and should not be interpreted as evidence of cognitive recovery or treatment-related improvement. Previous longitudinal studies have reported cognitive improvement following GFD adherence and mucosal healing [36,81]; however, such findings cannot be directly extrapolated to the present data. Alongside these data, a case series reported cognitive improvement or stabilization after gluten withdrawal in a subset of patients with cognitive decline temporally associated with biopsy-proven celiac disease, underscoring the potential clinical relevance of recognizing gluten-related cognitive involvement [78]. Another clinically relevant finding was that, among ND-CeD patients, MoCA scores were significantly correlated with WHOQOL-BREF-TR domains, including physical health, psychological health, and social relationships. This finding is consistent with broader literature suggesting that cognitive difficulties may affect quality of life through their impact on daily functioning and social participation in chronic disease populations [82,83]. By contrast, Croall et al. reported cognitive underperformance (particularly in verbal and visual memory) alongside selected SF-36 impairments, yet found no significant correlations between cognitive scores and QoL outcomes, indicating that these constructs may not consistently covary across samples. This discrepancy may reflect differences in cognitive measures (screening vs. domain-specific testing), QoL instruments (WHOQOL-BREF vs. SF-36), disease stage and symptom burden, and the extent to which psychosocial adaptation to celiac disease and the GFD moderates perceived quality of life [80]. However, pediatric data may indicate a partially different picture from adult findings. This discrepancy may reflect differences in cognitive measures (screening vs. domain-specific testing), QoL instruments (WHOQOL-BREF vs. SF-36), disease stage and symptom burden, and the extent to which psychosocial adaptation to celiac disease and the GFD moderates perceived quality of life [81].

Celiac disease has been associated with a higher burden of sleep problems, including insomnia symptoms, sleep-disordered breathing and poorer subjective sleep quality [24,27,84]. In line with these studies, we found that sleep quality was significantly poorer in ND-CeD patients than in control groups. This finding is consistent with prior adult data reporting impaired sleep quality in patients with CeD and with population-based evidence suggesting an increased burden of sleep-related problems around the time of diagnosis [27,85]. In newly diagnosed CeD, poorer sleep quality may be attributable to active disease and systemic factors. In particular, higher GI symptom severity may be associated with sleep disturbances [29,86,87]. Additionally, micronutrient deficiencies, particularly iron deficiency and iron deficiency anaemia, as well as folate and vitamin B12 deficiencies, are common at diagnosis and may contribute to fatigue and reduced functionality, which can further exacerbate perceived sleep disturbances [88]. Finally, psychological distress reported in CeD may contribute to sleep complaints during this period [14]. On the other hand, evidence on whether sleep outcomes differ between GFD-treated patients and controls in adults remains inconclusive. While several paediatric studies have reported improvements in sleep-related outcomes following GFD initiation [25,89,90], comparable evidence in adult populations is less consistent and appears insufficient to conclude that full normalisation has occurred. Indeed, studies in adults have reported no significant improvement in subjective sleep quality after adopting a GFD [26,27], suggesting that residual or subclinical sleep complaints may persist despite gluten withdrawal. In our study, although patients adhering to a GFD tended to have lower sleep quality scores than healthy controls, the between-group difference was not statistically significant. Given the cross-sectional design and the absence of pre–post assessment within the same individuals, no causal inference can be drawn regarding the effect of the GFD on sleep outcomes. It should also be noted that sleep quality in the present study was assessed using a single-item measure, which may not capture the multidimensional nature of sleep disturbance. To robustly evaluate the impact of a GFD on sleep in adults with celiac disease, prospective longitudinal studies incorporating validated sleep instruments and objective measures (e.g., actigraphy) are warranted. Potential confounders such as inadvertent gluten exposure, comorbidities, psychological distress, and micronutrient deficiencies should also be systematically assessed [24,27]. An association between MoCA scores and sleep quality was observed in the ND-CeD group at the uncorrected level; however, this did not survive FDR correction and should be regarded as a preliminary and hypothesis-generating observation. This association was not evident among patients following a GFD, although this should be interpreted cautiously given the modest subgroup sizes. To our knowledge, previous studies have not concurrently evaluated sleep quality and cognitive performance in adults with celiac disease; therefore, our findings provide preliminary evidence on this relationship. However, the cross-sectional design precludes formal mediation inference, and future longitudinal studies with repeated sleep and cognitive assessments are needed to test this potential pathway. Studies examining the relationship between sleep and cognitive performance in non-celiac GI diseases exist; however, the findings are inconsistent. While poor sleep indicators are associated with lower cognitive performance in some studies, this relationship has not been found in others [67,91,92]. Given the cross-sectional design, our findings specific to CeD should be interpreted with caution and confirmed in longitudinal studies covering the transition from untreated disease to a GFD. Clinically, healthcare providers managing newly diagnosed CeD patients may benefit from routinely evaluating sleep quality and cognitive complaints at diagnosis, as these may represent modifiable extraintestinal targets for follow-up. Given that a strict gluten-free diet remains the only established treatment for celiac disease, supporting dietary adherence, a primary responsibility of the dietitian, may also contribute to improvements in these outcomes as disease control is achieved.

In the present study, WHOQOL-BREF-TR scores were lower in ND-CeD patients than in healthy controls and GFD-CeD patients. Although GFD-CeD patients still showed lower General Health and Physical Health scores than controls, Psychological Health, Social Relationships, and Environment scores did not differ significantly from controls. These findings indicate a more favourable quality of life profile in GFD-CeD patients than in ND-CeD patients. However, as different individuals are compared at a single time point, this pattern should not be interpreted as evidence of within-person improvement following a GFD. The lower quality-of-life scores observed in ND-CeD patients should also be considered in the context of the broader symptom burden at diagnosis, including active gastrointestinal symptoms, fatigue, and psychological distress, which may collectively contribute to impaired well-being independently of celiac disease-specific mechanisms [93,94,95]. Consistent with our findings, previous studies have reported impaired quality of life in untreated and/or GFD–treated celiac patients compared with healthy individuals, with strict dietary adherence being associated with ‘near-normal’ levels in some cohorts [96,97]. A large pediatric study found that quality of life was largely similar between CeD patients and controls, and cognitive performance did not differ significantly in terms of accuracy, suggesting that both outcomes may follow a different trajectory in younger populations compared with the adult findings reported here [81]. However, other studies indicate that quality of life may remain lower than in controls despite adherence [98,99], potentially due to persistent extra-intestinal symptoms such as fatigue, depressive symptoms, mood changes, and pain [97,100,101]. In line with this heterogeneity, Croall et al. compared ND-CeD patients, patients on a long-term GFD, and controls, and found significant differences in specific subdomains (e.g., vitality and bodily pain), with lower vitality in ND-CeD patients and higher bodily pain in long-term treated patients [80]. Notably, in our cohort, selected quality-of-life domains were also associated with brain fog symptom burden and cognitive performance, supporting the relevance of neurocognitive complaints for patient-reported well-being. Given that quality of life in celiac disease is shaped by multiple interacting factors, including affordability and access to gluten-free products, cross-contamination concerns, continuous dietary vigilance, social restrictions and anxiety, adequacy of family/peer support, comorbidities, and access to regular follow-up, multidimensional strategies are warranted [20,93,97,102,103,104]. Beyond strengthening dietary adherence, interventions targeting psychological health and social support, reducing practical and economic barriers, and ensuring structured follow-up may help improve quality of life. Finally, quality of life is a key outcome in lifestyle interventions; in our study, regular physical activity was markedly lower in patients than in controls in the present study, which may have contributed to the observed quality-of-life differences. Promoting tailored physical activity may represent an additional opportunity to support both physical and mental well-being in CeD [20,93,97,102,103,104].

To explore possible neurobiological mechanisms underlying the observed cognitive and brain fog findings, we examined serum BDNF and S100B as exploratory markers potentially related to gut–brain axis signalling. No statistically detectable between-group differences were observed for either biomarker. Given the modest sample size and limited statistical power for biomarker comparisons, these null findings should not be interpreted as evidence of no difference but rather as reflecting an underpowered comparison in which smaller effects may not have been detectable. Evidence on peripheral BDNF alterations in CeD is heterogeneous [47,105]. In the study by Russo and colleagues, 19 celiac patients were followed on a GFD for at least 1 year, and both their serum BDNF values at the time of diagnosis and after following a GFD were compared with those of the control group. The study found that serum BDNF levels decreased by 12% in the group on a GFD compared to the time of diagnosis, but this did not reach statistical significance. Patients who followed a GFD were found to have significantly lower BDNF levels than the control group. Similar to our study, no significant difference was found between the control group and ND-CeD patients. The authors explained this decrease in BDNF levels in patients followed with a GFD by the stress experienced by patients due to dietary restrictions during the diet process [47]. Margoni et al. compared ND-CeD patients (n = 50), celiac patients who had been on a GFD for more than 1 year (n = 39), and a control group (n = 36) in terms of serum BDNF levels. Both ND-CeD patients and patients followed up on a GFD had significantly higher BDNF levels compared to the control group. When BDNF levels were measured again after 1 year of GFD in 8 children among the ND-CeD, no difference was found in BDNF levels. The authors interpreted the increased BDNF levels in patients as a compensatory or protective response to inflammatory reactions in the intestines and ongoing stress [105]. These discrepant findings likely reflect differences in study design, sample characteristics, assay methodology, and age-related factors (pediatric vs. adult cohorts). Taken together, the literature on peripheral BDNF in CeD remains inconsistent, and our null finding does not allow firm conclusions. Larger, well-powered longitudinal studies in adult cohorts are needed to determine whether serum BDNF varies meaningfully with disease activity and GFD adherence in CeD.

To our knowledge, no studies have evaluated circulating S100B levels specifically in CeD. In our study, although S100B levels were found to be high in the control group compared to other groups, there was no significant difference between groups. S100B is a pleiotropic calcium-binding protein with concentration-dependent extracellular effects, ranging from neurotrophic signalling at low levels to potentially deleterious actions at higher concentrations [106]. Esposito et al. demonstrated that enteric glial–derived S100B is upregulated in duodenal mucosa in untreated CeD and is mechanistically linked to NO signalling, as evidenced by parallel increases in iNOS expression and nitrite production and by inhibition of gliadin-induced responses with an anti-S100B antibody [107]. This study indicates that S100B is associated with a pathway that is particularly activated in the mucosal compartment in CeD [107]. Consistent with this, data also exist suggesting that S100B in the systemic circulation may not always reflect disease activity: Celikbilek et al. reported that serum S100B was significantly lower in ulcerative colitis patients compared to controls; furthermore, they found no correlation between serum S100B and disease activity/duration, and no difference between active disease and remission [108]. Taken together, available evidence suggests that S100B may be relevant to CeD-related mucosal and glial pathways; however, circulating S100B may not directly reflect mucosal compartment activity or cognitive outcomes. Therefore, the absence of a statistically detectable difference in serum S100B in the present study should be interpreted cautiously and does not exclude a potential compartment-specific role of S100B in CeD. Future adequately powered longitudinal studies should evaluate serum S100B together with mucosal markers and well-defined cognitive endpoints to clarify its relevance in CeD-related neurocognitive symptoms.

Evidence on circulating IL-6 in celiac disease is inconsistent, and we similarly found no significant between-group differences in serum IL-6. Consistent with our findings, Tack et al. reported comparable serum IL-6 concentrations in ND-CeD patients and those on a GFD, whereas markedly higher IL-6 levels were observed in refractory celiac disease, suggesting that circulating IL-6 may not clearly distinguish uncomplicated disease from treated CeD but may increase in more severe or complicated phenotypes (e.g., RCDII and EATL). In the same report, IL-8 and IL-17 appeared more responsive to active disease status, supporting the notion that broader cytokine profiles may be more informative than IL-6 alone [109]. Similarly, Aljamrawy et al. (2024) found only a modest elevation of IL-6 in active CeD versus controls, while IL-6 did not differ between active and GFD groups [110]. Differences across studies may also reflect variation in the biological compartment assessed. For example, Asri et al. reported increased IL-6 gene expression in peripheral blood mononuclear cells (PBMCs) in ND-CeD patients compared with controls; however, transcriptional changes in immune cells do not necessarily translate into proportional changes in circulating protein levels [111]. Further underscoring heterogeneity, a case–control study from Iran reported an inverse association between serum IL-6 and the odds of celiac disease [112]. Age and disease context may also contribute: in paediatric cohorts, serum IL-6 has been reported to be higher in ND-CeD children than in controls [113], while in adults, IL-6 was higher in active CeD than in controls and decreased in those on a GFD [41]. Collectively, these findings suggest that serum IL-6 alone may have limited sensitivity in uncomplicated celiac disease. Future work may benefit from incorporating broader cytokine panels (e.g., IL-8/IL-17) and immune activation markers (e.g., sCD25) and/or local readouts such as duodenal mucosal expression rather than relying solely on serum IL-6. In this context, an experimental study using duodenal biopsies and patient-derived organoids has shown increased enterocyte IL-6 (and IL-1β) expression, which can persist even in potential celiac disease and in patients on a GFD, alongside sustained activation of inflammatory signalling pathways (e.g., NF-κB/ERK) and heightened responsiveness to gliadin- and TLR-mediated stimuli [114]. Our study did not detect significant between-group differences in serum NO, suggesting that circulating NO may have limited discriminatory value in our cohort and should be interpreted cautiously. In contrast, serum NO has been described as higher at diagnosis and lower after one year on a GFD, correlating with histologic severity in pediatric [115]. Several studies using local or non-serum compartments have reported clearer signals: adult duodenal enterocytes show increased iNOS activity/expression in untreated disease with a decrease on a GFD [116], rectal gluten challenge elicits a marked rise in local NO following earlier granulocyte activation [117], and ex vivo duodenal biopsies demonstrate increased iNOS/nitrite (with S100B involvement) in untreated disease [107]. In paediatric cohorts, urinary nitrite/nitrate and, in some reports, serum NO have been elevated at diagnosis and decreased after a GFD [118]. In our study, the absence of significant between-group differences in serum NO does not rule out a role for the NO pathway in CeD pathophysiology; rather, it suggests that circulating measurements may have limited discriminatory value as a biomarker in this cohort. Adult serum data remain limited and heterogeneous, and potential differences may be obscured by methodological variability (including differences in NO/NOx assays and pre-analytical conditions), comorbid inflammatory states, and age-related factors. Therefore, our serum NO findings should be interpreted cautiously and warrant confirmation in larger studies using standardised assessment of NO metabolites alongside mucosal markers of inflammation [116,119]. These observations, both for IL-6 and NO, support the possibility that local mucosal inflammation is more readily detected than by systemic serum measures. We observed no between-group differences in circulating TLR4 measured in serum. Evidence on TLR4 in celiac disease is heterogeneous: while several studies report altered TLR expression at the mucosal or cellular level [120,121,122], genetic studies have not supported an association between common TLR4 variants (e.g., Asp299Gly/Thr399Ile) and celiac disease [123,124,125]. Differences in the biological compartment and the analyte may contribute to discrepant findings.

### Strength Limitations and Future Directions

Strengths of this study include its comparative design with clinically defined CeD groups and healthy controls, and the concurrent assessment of brain fog symptoms, cognitive performance, sleep quality, quality of life, and serum biomarkers within a single study protocol. This multidomain approach provides a comprehensive, patient-centred preliminary evaluation of extraintestinal outcomes in CeD. Efforts were made to recruit healthy controls with a similar age and sex distribution to the CeD groups, and the groups did not differ significantly in age or sex distribution.

Several limitations should be acknowledged. First, the modest sample size limits statistical power, particularly for serum biomarker analyses, subgroup correlations, and exploratory regression models. Although the sample size is comparable to previous pilot studies examining cognitive outcomes in CeD [36,80,126], the findings should be interpreted as preliminary and hypothesis-generating rather than confirmatory. The hierarchical regression analyses should also be interpreted cautiously because the number of predictors relative to the total sample size may have limited model stability and precision. Similarly, the serum biomarker analyses may have had limited sensitivity to detect small-to-moderate between-group differences in BDNF, S100B, IL-6, NO, and TLR4. Therefore, non-significant biomarker findings should be interpreted as an absence of statistically detectable differences in this sample rather than as evidence of no biological difference.

Second, although the groups did not differ significantly in age or sex distribution, differences were observed in income status, smoking, and regular physical activity. These variables may influence cognitive performance, sleep quality, quality of life, inflammatory status, and biomarker levels. In clinical observational studies, demographic and lifestyle factors are often closely interrelated with socioeconomic conditions, disease status, symptom burden, and health-related behaviors, making complete control at the recruitment stage challenging [127,128]. In the present study, regular physical activity was operationalized according to whether participants met recommended activity levels, rather than as an indicator of participation in vigorous exercise per se [129]. Although selected demographic and lifestyle covariates were considered in exploratory regression analyses, the modest sample size limited the extent to which all potential confounders could be simultaneously adjusted for without increasing the risk of overfitting. In addition, controls were recruited using convenience-based community sampling and were not individually matched for socioeconomic or lifestyle characteristics. Although comprehensive exclusion criteria helped minimize confounding from comorbid conditions, medication use, and other health-related factors, residual confounding related to socioeconomic status and lifestyle cannot be excluded. Future studies should consider individual matching or stratified recruitment strategies.

Micronutrient and anemia-related factors should also be considered when interpreting cognitive performance, sleep quality, fatigue, and brain fog symptoms. Although ferritin, folate, and vitamin B12 were examined in supplementary analyses, the modest sample size limited the extent to which these variables could be comprehensively adjusted for in the main models. Therefore, residual confounding by micronutrient status and anemia-related parameters cannot be excluded [130,131].

Third, gluten-free diet adherence in the GFD-CeD group was based primarily on self-report and the duration criterion of at least two years. Although available serological markers were considered where available, inadvertent gluten exposure was not systematically assessed, and mucosal healing was not confirmed histologically. Objective assessment of gluten-free diet adherence, such as repeated measurements of gluten immunogenic peptides in urine or stool, may help future studies better characterize recent gluten exposure, while accounting for the short detection window and timing-dependent nature of these tests [50,51]. Furthermore, GFD duration was not further stratified; therefore, potential differences between patients with shorter versus longer treatment duration may have been masked, contributing to within-group heterogeneity in the GFD-CeD group.

Fourth, sleep quality was assessed using the SQS. Although the SQS provides a practical global measure of perceived sleep quality and has demonstrated adequate psychometric properties, including moderate-to-strong associations with Pittsburgh Sleep Quality Index scores, it does not capture the multidimensional nature of sleep as comprehensively as validated multi-item instruments, nor does it provide the objective assessment afforded by actigraphy or polysomnography [25,132]. Therefore, sleep-related findings should be interpreted cautiously. Finally, the cross-sectional design precludes causal inference. The between-group differences observed in this study reflect associations at a single time point and should not be interpreted as evidence of treatment-related improvement, cognitive recovery, or causal effects of a gluten-free diet.

Future studies should use larger, prospectively designed cohorts with repeated within-subject assessments from diagnosis through at least 12–24 months of follow-up to better characterize the trajectory of brain fog symptoms, cognitive performance, sleep quality, and quality of life in relation to GFD adherence and disease activity [36,49]. Incorporating objective measures of gluten exposure, serological follow-up, and, where feasible, histological assessment of mucosal recovery would strengthen interpretation of diet-related findings. Given prior reports of white matter abnormalities in CeD, neuroimaging approaches such as MRI, together with neurophysiological tools such as EEG, may provide complementary structural and functional insight into neurocognitive involvement [12,16]. Future studies should also incorporate comprehensive sleep assessment, including validated multi-item sleep questionnaires and objective monitoring of sleep–wake patterns using actigraphy. In addition, assessment of micronutrient status, anemia-related parameters, and inflammatory markers, together with stool-based microbiome profiling, microbial metabolites such as short-chain fatty acids, bile acids, and tryptophan-derived metabolites, and markers of intestinal barrier integrity or microbial translocation, may help clarify biological links between intestinal changes, sleep, and neurocognitive outcomes in CeD [9,133].

## 5. Conclusions

In this comparative cross-sectional observational study, newly diagnosed celiac disease patients showed a higher burden of brain fog symptoms, poorer cognitive performance, worse sleep quality, and lower quality-of-life scores compared with healthy controls. GFD-CeD patients generally showed intermediate or more favorable scores than ND-CeD patients in some outcomes, with several findings appearing closer to control values; however, these differences should not be interpreted as evidence of improvement caused by a gluten-free diet, as different individuals were compared at a single time point. These findings suggest that brain fog, cognitive complaints, sleep quality, and quality of life warrant attention from gastroenterologists, dietitians, and other relevant healthcare providers at the time of diagnosis and during clinical follow-up. Longitudinal studies with larger samples and objective assessment of gluten-free diet adherence, disease activity, and sleep quality are needed to clarify whether and how these outcomes change over time.

## Figures and Tables

**Table 1:** General characteristics of the participants.

		Groups						*p*
		ND-CeD Patients (*n* = 18)		GFD-CeD (*n* = 17)		Controls (*n* = 27)		
		n	%	n	%	n	%	
Age	≤30	8	44.4	9	52.9	14	51.9	0.856
	31–45	5	27.8	6	35.3	8	29.6	
	≥46	5	27.8	2	11.8	5	18.5	
Gender	Female	13	72.2	16	94.1	20	74.1	0.212
	Male	5	27.8	1	5.9	7	25.9	
Education level	Primary education	3	16.7	2	11.8	3	11.1	0.063
	High school	7	38.9	11	64.7	5	18.5	
	Bachelor’s degree	7	38.9	3	17.6	14	51.9	
	Postgraduate	1	5.6	1	5.9	5	18.5	
Income level	Below the national minimum wage	2	11.1	1	5.9	4	14.8	<0.001 ***
	Equal to the national minimum wage	11	61.1	5	29.4	1	3.7	
	Above the national minimum wage	5	27.8	11	64.7	22	81.5	
Smoking	Yes	5	27.8	8	47.1	—	—	<0.001 ***
	No	13	72.2	9	52.9	27	100.0	
Alcohol	Yes	—	—	2	11.8	—	—	0.072
	No	18	100.0	15	88.2	27	100.0	
Regular physical activity (≥150 min/week, ≥3 days/week)	Yes	1	5.6	1	5.9	12	44.4	0.001 **
	No	17	94.4	16	94.1	15	55.6	

Data are presented as n (%). *p* values were calculated using chi-square test or Fisher’s exact test where appropriate. ND-CeD, newly diagnosed celiac disease; GFD-CeD, celiac disease on gluten-free diet. ** *p* < 0.01; *** *p* < 0.001.

**Table 2:** Group differences in sleep quality and MoCA-defined cognitive impairment.

		Groups						*p*
		ND-CeD (*n* = 18)		GFD-CeD (*n* = 17)		Controls (*n* = 27)		
		n	%	n	%	n	%	
Sleep Quality	Very poor	1	5.6	-	-	-	-	0.015 *
	Poor	6	33.3	2	11.8	3	11.1	
	Fair	8	44.4	9	52.9	6	22.2	
	Good	3	16.7	5	29.4	17	63.0	
	Excellent	-	-	1	5.9	1	3.7	
MoCA	Possible cognitive impairment	10	55.6	4	23.5	1	3.7	<0.001 ***
	No cognitive impairment	8	44.4	13	76.5	26	96.3	

Data are presented as n (%). *p* values were calculated using chi-square test or Fisher’s exact test where appropriate. * *p* < 0.05; *** *p* < 0.001. ND-CeD, newly diagnosed celiac disease; GFD-CeD, celiac disease on gluten-free diet; MoCA, Montreal Cognitive Assessment.

**Table 3:** Comparison of brain fog symptoms, cognitive performance, sleep quality, and quality of life among the study groups.

	ND-CeD (*n* = 18) (1)	GFD-CeD (*n* = 17) (2)	Controls (*n* = 27) (3)	F	*p*	Post Hoc	η^2^
Brain Fog Scale (BFS)	60.94 ± 20.32	51.35 ± 16.76	47.25 ± 12.61	3.85	0.027 *	1 > 3	0.116
BFS-Mental fatigue	23.77 ± 9.07	19.47 ± 7.85	19.07 ± 7.37	2.06	0.136		0.065
BFS-Impaired cognitive acuity	18.77 ± 8.74	15.05 ± 5.80	13.66 ± 3.38	3.97	0.024 *	1 > 3	0.119
BFS-Confusion	18.38 ± 6.44	16.82 ± 4.79	14.51 ± 4.63	3.05	0.055		0.094
BFSS	51.66 ± 26.17	36.47 ± 22.62	23.33 ± 18.81	8.85	<0.001 ***	1 > 3	0.231
The Montreal Cognitive Assessment (MoCA)	20.05 ± 4.49	22.94 ± 3.50	25.07 ± 3.94	8.51	0.001 **	3 > 1	0.224
Single-Item Sleep Quality Scale (SQS)	4.5 ± 2.35	5.94 ± 2.01	6.85 ± 1.87	7.03	0.002 **	3 > 1	0.192
WHOQOL-BREF- TR Physical Health	53.37 ± 16.41	66.60 ± 18.38	78.31 ± 16.87	11.46	0.001 **	3 > 1	0.280
WHOQOL-BREF- TR Psychological Health	49.77 ± 11.65	66.67 ± 12.67	68.21 ± 17.95	9.11	0.001 **	2 > 1 3 > 1	0.236
WHOQOL-BREF- TR Social Relationships	43.52 ± 17.52	67.16 ± 18.04	68.83 ± 18.58	11.86	0.001 **	2 > 1 3 > 1	0.287
WHOQOL-BREF- TR Environment	62.85 ± 18.84	72.43 ± 14.29	75.69 ± 13.01	3.93	0.025 *	3 > 1	0.118

Note: WHOQOL-BREF domain scores were transformed to a 0–100 scale, with higher scores indicating better quality of life. Group comparisons were performed using one-way ANOVA, followed by post hoc analyses for variables showing significant differences. Post hoc comparisons were conducted using Bonferroni or Tamhane’s T2 tests depending on homogeneity of variances. * *p* < 0.05; ** *p* < 0.01; *** *p* < 0.001. η^2^, eta-squared effect size. Normality was assessed using the Shapiro–Wilk test; for variables with normality violations, Kruskal–Wallis tests yielded consistent results. BFS, Brain Fog Scale; BFSS, Brain Fog Severity Score; MoCA, Montreal Cognitive Assessment; SQS, Single-Item Sleep Quality Scale; WHOQOL-BREF-TR, World Health Organization Quality of Life Questionnaire-Brief Form-Turkish.

**Table 4:** Within-group correlations between brain fog symptoms, cognitive performance, sleep quality, and quality of life in ND-CeD patients (*n* = 18) and GFD-CeD patients (*n* = 17).

Variable	ND-CeD BFS r (*p*)	q	ND-CeD MoCA r (*p*)	q	GFD-CeD BFS r (*p*)	q	GFD-CeD MoCA r (*p*)	q
SQS	−0.340 (0.167)	0.217	0.522 (0.026)	0.068	−0.304 (0.235)	0.382	0.355 (0.162)	0.362
WHOQOL-BREF-TR General Health	−0.146 (0.563)	0.665	0.405 (0.095)	0.137	−0.260 (0.314)	0.454	0.351 (0.167)	0.362
WHOQOL-BREF-TR Physical Health	−0.482 (0.043)	0.080	0.628 (0.005)	0.016 *	−0.521 (0.032)	0.139	−0.056 (0.832)	0.983
WHOQOL-BREF-TR Psychological Health	−0.917 (<0.001)	0.004 *	0.750 (<0.001)	0.004 *	−0.566 (0.018)	0.139	0.330 (0.195)	0.362
WHOQOL-BREF-TR Social Relationships	−0.449 (0.061)	0.099	0.481 (0.043)	0.080	−0.468 (0.058)	0.189	0.172 (0.508)	0.660
WHOQOL-BREF-TR Environment	−0.066 (0.796)	0.799	−0.065 (0.799)	0.799	−0.018 (0.946)	0.994	0.534 (0.027)	0.139
MoCA	−0.693 (0.001)	0.004 *	—	—	0.002 (0.994)	0.994	—	—

Values are Pearson correlation coefficients, with unadjusted two-sided *p* values shown in parentheses. Benjamini–Hochberg FDR adjustment was applied separately to the 13 correlations tested within each study group. q values represent FDR-adjusted *p* values. * q < 0.05. Given the small subgroup sizes (ND-CeD: n = 18; GFD-CeD: n = 17), these exploratory associations should be interpreted cautiously and regarded as hypothesis-generating.

**Table 5:** Serum biomarker levels across study groups.

Serum Parameters	Statistic	ND-CeD (*n* = 18)	GFD-CeD (*n* = 17)	Controls (*n* = 27)	H	*p*	η^2^H
BDNF (ng/mL)	Mean ± SD Median (IQR)	1.42 ± 0.72 1.22 (0.96–1.62)	1.31 ± 0.46 1.11 (1.05–1.35)	1.60 ± 0.73 1.28 (1.10–2.05)	1.665	0.435	0.027
S100B (ng/L)	Mean ± SD Median (IQR)	331.37 ± 167.69 288.50 (230.91–366.08)	320.71 ± 104.63 297.97 (267.01–319.62)	363.18 ± 151.90 325.52 (251.40–443.22)	1.535	0.464	0.025
TLR4 (ng/mL)	Mean ± SD Median (IQR)	1.73 ± 0.77 1.49 (1.19–1.84)	1.43 ± 0.33 1.40 (1.21–1.47)	1.69 ± 0.70 1.43 (1.08–2.19)	0.822	0.663	0.013
IL-6 (ng/L)	Mean ± SD Median (IQR)	58.09 ± 30.50 49.32 (35.48–83.01)	49.40 ± 22.34 39.48 (34.88–64.22)	56.10 ± 23.24 51.70 (39.80–72.24)	1.868	0.393	0.031
NO (µmol/L)	Mean ± SD Median (IQR)	73.50 ± 44.68 57.89 (46.03–81.99)	61.84 ± 28.13 53.99 (48.20–58.61)	72.33 ± 31.01 64.67 (49.72–98.42)	1.726	0.422	0.028

Note. Data are presented as mean ± SD and median (IQR). *p*-values were obtained using the Kruskal–Wallis test. Effect size is presented as Kruskal–Wallis eta-squared based on the H statistic. No statistically detectable between-group differences. Given the limited statistical power for biomarker comparisons (sensitivity analysis: Cohen’s f = 0.404), null findings should not be interpreted as evidence of no difference; smaller or clinically meaningful differences cannot be excluded. Pairwise Hedges’ g effect sizes with 95% confidence intervals are provided in Supplementary Table S1. ND-CeD = newly diagnosed celiac disease; GFD-CeD = celiac disease following a gluten-free diet; BDNF = brain-derived neurotrophic factor; S100B = S100 calcium-binding protein B; TLR4 = toll-like receptor 4; IL-6 = interleukin-6; NO = nitric oxide.

**Table 6:** Hierarchical multiple linear regression analysis identifying factors associated with MoCA total score.

Variable	Model 1 B (95% CI)	*p*	Model 2 B (95% CI)	*p*
Age (years)	−0.19 (−0.27, −0.10)	<0.001 ***	−0.16 (−0.25, −0.08)	<0.001 ***
Income (above minimum wage)	4.68 (1.13, 8.23)	0.011 *	3.93 (0.39, 7.47)	0.030 *
Income (at minimum wage)	1.04 (−2.56, 4.64)	0.567	2.03 (−1.57, 5.63)	0.263
Physical activity (yes)	−1.50 (−3.89, 0.89)	0.214	−0.54 (−3.07, 1.98)	0.668
ND-CeD vs. control	—	—	−3.46 (−6.37, −0.56)	0.020 *
GFD-CeD vs. control	—	—	−1.69 (−4.24, 0.87)	0.191
Model fit				
R^2^	0.325		0.389	
Adjusted R^2^	0.278		0.322	
ΔR^2^	—		0.064	
Model *p* value	<0.001		—	
*p* for F change	—		0.066	

Note: Values are presented as unstandardized regression coefficients (B) with 95% confidence intervals (CI). Model 1 included age, income status, and regular physical activity. Model 2 additionally included group status, with the control group as the reference category. Reference categories were income below the national minimum wage, no regular physical activity, and the control group. * *p* < 0.05; *** *p* < 0.001. ND-CeD, newly diagnosed celiac disease; GFD-CeD, celiac disease on a gluten-free diet; MoCA, Montreal Cognitive Assessment.

## Data Availability

The data supporting the findings of this study are available from the corresponding author upon reasonable request.

## Supplementary Materials

The following supporting information can be downloaded at https://www.mdpi.com/article/10.3390/nu18142365/s1: Table S1: Pairwise Hedges’ g effect sizes with 95% confidence intervals for serum biomarker comparisons; Table S2: Hierarchical multiple linear regression analysis identifying factors associated with sleep quality scores; Table S3: Exploratory hierarchical regression analysis for Brain Fog Scale scores; Table S4: Exploratory hierarchical regression analyses for WHOQOL-BREF-TR domain scores; Table S5: Serum micronutrient levels across study groups]; Table S6: Sensitivity analysis: Hierarchical multiple linear regression for MoCA total scores additionally including micronutrient parameters as covariates; Figure S1: Boxplots illustrating the distribution of serum BDNF and S100B levels across study groups (ND-CeD, n = 18; GFD-CeD, n = 17; controls, n = 27); Figure S2: Boxplots illustrating the distribution of serum IL-6, NO, and TLR-4 levels across study groups (ND-CeD, n = 18; GFD-CeD, n = 17; controls, n = 27).

## Abbreviations

The following abbreviations are used in this manuscript:

CeD	Celiac disease
ND-CeD	Newly diagnosed patients with celiac disease
GFD-CeD	Patients with CeD on a gluten-free diet
BFS	Brain Fog Scale
BFSS	Brain Fog Severity Score
MoCA	Montreal Cognitive Assessment
SQS	Single-Item Sleep Quality Scale
WHOQOL-BREF-TR	World Health Organization Quality of Life Questionnaire-Brief Form-TR
GBA	Gut–brain axis
GI	Gastrointestinal
