# Supplementary material for: Beyond the Gut: Brain Fog, Sleep Quality, Cognitive Function and Quality of Life in Celiac Disease
Source: Nutrients. 2026 Jul 19;18(14):2365. doi: 10.3390/nu18142365 (PMC13414575; doi:10.3390/nu18142365)
Supplement: Supplementary file 1 [file nutrients-18-02365-s001.zip › nutrients-4352322-supplementary.pdf]

Supplementary Material

Supplementary Table S1. Pairwise Hedges' g effect sizes with 95% confidence intervals for serum biomarker comparisons.

Biomarker	Comparison	Hedges' g	95% CI
**BDNF**	ND-CeD vs Control	−0.244	(−0.843, 0.355)
**BDNF**	GFD-CeD vs Control	−0.451	(−1.066, 0.163)
**BDNF**	ND-CeD vs GFD-CeD	0.184	(−0.480, 0.849)
**IL-6**	ND-CeD vs Control	0.075	(−0.522, 0.671)
**IL-6**	GFD-CeD vs Control	−0.287	(−0.897, 0.323)
**IL-6**	ND-CeD vs GFD-CeD	0.316	(−0.351, 0.984)
**S100B**	ND-CeD vs Control	−0.197	(−0.795, 0.401)
**S100B**	GFD-CeD vs Control	−0.307	(−0.917, 0.303)
**S100B**	ND-CeD vs GFD-CeD	0.074	(−0.589, 0.737)
**TLR-4**	ND-CeD vs Control	0.046	(−0.550, 0.643)
**TLR-4**	GFD-CeD vs Control	−0.433	(−1.047, 0.180)
**TLR-4**	ND-CeD vs GFD-CeD	0.480	(−0.193, 1.153)
**NO**	ND-CeD vs Control	0.031	(−0.565, 0.627)
**NO**	GFD-CeD vs Control	−0.344	(−0.955, 0.267)
**NO**	ND-CeD vs GFD-CeD	0.303	(−0.364, 0.970)

Note. Hedges' g was calculated as a complementary descriptive effect size measure. Although serum biomarkers did not fully meet normality assumptions, Hedges' g is reported as a widely used descriptive effect size to allow readers to evaluate the magnitude of pairwise differences. All 95% confidence intervals include zero, consistent with the non-significant Kruskal-Wallis results. Small-to-medium effect sizes were observed for some comparisons, further supporting the interpretation that the study may have been insufficiently powered to detect realistic biomarker differences. BDNF, brain-derived neurotrophic factor; IL-6, interleukin-6; NO, nitric oxide; S100B, S100 calcium-binding protein B; TLR-4, toll-like receptor 4; ND-CeD, newly diagnosed celiac disease; GFD-CeD, celiac disease on a gluten-free diet.

Supplementary Table S2. Hierarchical multiple linear regression analysis identifying factors associated with sleep quality scores.

Variable	Model 1 B (95% CI)	p	Model 2 B (95% CI)	p
**Age (years)**	−0.05 (−0.10, 0.00)	0.058	−0.03 (−0.08, 0.02)	0.183
**Income (above minimum wage)**	1.25 (−0.79, 3.28)	0.224	0.78 (−1.24, 2.80)	0.442
**Income (at minimum wage)**	−0.30 (−2.36, 1.76)	0.775	0.26 (−1.80, 2.31)	0.802
**Physical activity (yes)**	−0.51 (−1.88, 0.86)	0.457	0.00 (−1.44, 1.44)	0.999
**ND-CeD vs control**	—	—	−2.00 (−3.66, −0.34)	**0.019**
**GFD-CeD vs control**	—	—	−0.83 (−2.29, 0.63)	0.262
**Model fit**				
**R^2**	0.136		0.219	
**Adjusted R^2**	0.075		0.134	
**ΔR^2**	—		0.083	
**Model p value**	0.076		—	
**p for F change**	—		0.062	

Note. Values are presented as unstandardized regression coefficients (B) with 95% confidence intervals (CI). Model 1 included age, income status, and regular physical activity. Model 2 additionally included group status, with the control group as the reference category. Reference categories were income below the national minimum wage, no regular physical activity, and the

control group. *p < 0.05. ND-CeD, newly diagnosed celiac disease; GFD-CeD, celiac disease on a gluten-free diet; SQS, Single-Item Sleep Quality Scale.

Supplementary Table S3. Exploratory hierarchical regression analysis for Brain Fog Scale scores.

Variable	Model 1 B (95% CI)	p	Model 2 B (95% CI)	p
**Age (years)**	0.13 (−0.25, 0.51)	0.507	0.05 (−0.34, 0.44)	0.800
**Income (above minimum wage)**	−4.09 (−19.79, 11.62)	0.604	−1.31 (−17.39, 14.77)	0.871
**Income (at minimum wage)**	5.04 (−10.87, 20.95)	0.528	2.74 (−13.61, 19.08)	0.739
**Physical activity (yes)**	6.26 (−4.32, 16.83)	0.241	4.53 (−6.93, 15.99)	0.432
**ND-CeD vs control**	—	—	9.52 (−3.67, 22.72)	0.154
**GFD-CeD vs control**	—	—	1.39 (−10.23, 13.01)	0.811
**Model fit**				
**Adjusted R^2**	0.031		0.039	
**R^2**	0.095		0.133	
**ΔR^2**	—		0.039	
**Model p value**	0.218		—	
**p for F change**	—		0.302	

Note: Values are presented as unstandardized regression coefficients (B) with 95% confidence intervals (CI). Model 1 included age, income status, and regular physical activity. Model 2 additionally included group status, with the control group as the reference category. Reference categories were income below the national minimum wage, no regular physical activity, and the control group. Neither model was statistically significant, and no covariates or group contrasts were statistically significant in the fully adjusted model. These analyses are reported for transparency and should be interpreted as exploratory given the modest sample size. BFS, Brain Fog Scale; ND-CeD, newly diagnosed celiac disease; GFD-CeD, celiac disease on a gluten-free diet.

Supplementary Table S4. Exploratory hierarchical regression analyses for WHOQOL-BREF-TR domain scores.

Domain/Variable	Model 1 B (95% CI)	p	Model 2 B (95% CI)	p
**Physical Health**				
**Age (years)**	−0.10 (−0.22, 0.02)	0.112	−0.05 (−0.17, 0.07)	0.397
**Income (above minimum wage)**	1.45 (−3.63, 6.52)	0.570	−0.12 (−4.85, 4.61)	0.960
**Income (at minimum wage)**	−2.12 (−7.26, 3.02)	0.412	−0.01 (−4.81, 4.80)	0.998
**Physical activity (yes)**	−1.01 (−4.43, 2.41)	0.556	1.05 (−2.32, 4.42)	0.535
**ND-CeD vs control**	—	—	**−7.33 (−11.21, −3.45)**	**<0.001*****
**GFD-CeD vs control**	—	—	−3.67 (−7.09, −0.26)	0.036*
**Psychological Health**				
**Age (years)**	−0.06 (−0.15, 0.03)	0.170	−0.03 (−0.11, 0.06)	0.529
**Income (above minimum wage)**	0.97 (−2.71, 4.65)	0.600	−0.35 (−3.87, 3.18)	0.845
**Income (at minimum wage)**	−0.93 (−4.66, 2.80)	0.618	−0.06 (−3.64, 3.52)	0.972
**Physical activity (yes)**	−1.87 (−4.35, 0.61)	0.136	−1.35 (−3.86, 1.16)	0.287
**ND-CeD vs control**	—	—	**−3.98 (−6.87, −1.10)**	**0.008**
**GFD-CeD vs control**	—	—	0.12 (−2.42, 2.67)	0.923
**Social Relationships**				

**Age (years)**	−0.04 (−0.10, 0.01)	0.136	−0.02 (−0.07, 0.03)	0.474
**Income (above minimum wage)**	1.03 (−1.24, 3.30)	0.367	0.17 (−1.98, 2.31)	0.876
**Income (at minimum wage)**	−0.66 (−2.96, 1.64)	0.568	−0.10 (−2.28, 2.08)	0.930
**Physical activity (yes)**	−0.97 (−2.50, 0.56)	0.211	−0.63 (−2.16, 0.90)	0.410
**ND-CeD vs control**	—	—	**−2.60 (−4.36, −0.84)**	**0.004**
**GFD-CeD vs control**	—	—	0.11 (−1.44, 1.66)	0.890
**Environment**				
**Age (years)**	−0.04 (−0.16, 0.07)	0.457	−0.00 (−0.12, 0.11)	0.939
**Income (above minimum wage)**	0.45 (−4.44, 5.33)	0.855	−0.92 (−5.71, 3.88)	0.702
**Income (at minimum wage)**	−0.23 (−5.17, 4.72)	0.927	1.13 (−3.75, 6.00)	0.645
**Physical activity (yes)**	−1.22 (−4.51, 2.07)	0.461	−0.08 (−3.50, 3.34)	0.963
**ND-CeD vs control**	—	—	**−5.21 (−9.14, −1.27)**	**0.010**
**GFD-CeD vs control**	—	—	−1.46 (−4.92, 2.01)	0.403

Model fit:

Domain	Model 1 Adj R^2	Model 2 Adj R^2	ΔR^2	p for F change
**Physical Health**	0.053	0.222	0.183	0.002
**Psychological Health**	0.056	0.183	0.145	0.007
**Social Relationships**	0.084	0.228	0.160	0.003
**Environment**	−0.043	0.048	0.116	0.031

Note: Values are presented as unstandardized regression coefficients (B) with 95% confidence intervals (CI). Separate exploratory hierarchical regression models were conducted for each WHOQOL-BREF-TR domain because the instrument does not yield a validated total score. Model 1 included age, income status, and regular physical activity. Model 2 additionally included group status, with the control group as the reference category. Reference categories were income below the national minimum wage, no regular physical activity, and the control group. *p < 0.05; **p < 0.01; ***p < 0.001. These exploratory models should be interpreted cautiously given the modest sample size. WHOQOL-BREF-TR, World Health Organization Quality of Life Questionnaire-Brief Form–Turkish; ND-CeD, newly diagnosed celiac disease; GFD-CeD, celiac disease on a gluten-free diet.

Supplementary Table S5. Serum micronutrient levels across study groups.

	ND-CeD (n=18)	GFD-CeD (n=17)	Controls (n=27)	Test	p	Post-hoc
**Ferritin (ng/mL)**	17.44±9.21	17.38±9.00	34.42±23.12	H=8.526	0.014*	3>1, 3>2
**Folate (ng/mL)**	8.18±2.81	6.62±1.87	7.80±3.48	H=1.912	0.384	—
**B12 (pg/mL)**	279.67±76.09	353.94±78.90	329.04±77.02	F=4.265	0.019*	2>1

Note. Data are presented as mean ± SD. Ferritin and folate were compared using the Kruskal–Wallis test because the assumptions for parametric analysis were not met. Group numbers: 1 = ND-CeD, 2 = GFD-CeD, 3 = Control. * p < 0.05 ND-CeD, newly diagnosed celiac disease; GFD-CeD, celiac disease on a gluten-free diet.

Supplementary Table S6. Sensitivity analysis: Hierarchical multiple linear regression for MoCA total scores additionally including micronutrient parameters as covariates

Variable	Model 1 B (95% CI)	p	Model 2 B (95% CI)	p

**Age (years)**	−0.18 (−0.27, −0.08)	<0.001***	−0.16 (−0.25, −0.07)	0.001**
**Income (above minimum wage)**	4.28 (0.43, 8.13)	0.030*	3.99 (0.19, 7.78)	0.040*
**Income (at minimum wage)**	0.86 (−2.87, 4.60)	0.646	2.00 (−1.80, 5.80)	0.295
**Physical activity (yes)**	−1.43 (−3.88, 1.02)	0.248	−0.58 (−3.17, 2.01)	0.656
**Ferritin (ng/mL)**	0.02 (−0.03, 0.08)	0.412	0.00 (−0.06, 0.06)	0.887
**Folate (ng/mL)**	−0.12 (−0.46, 0.23)	0.509	−0.09 (−0.44, 0.26)	0.597
**Vitamin B12 (pg/mL)**	0.00 (−0.01, 0.01)	0.998	−0.00 (−0.02, 0.01)	0.727
**ND-CeD vs control**	—	—	−3.42 (−6.59, −0.25)	0.035*
**GFD-CeD vs control**	—	—	−1.64 (−4.54, 1.26)	0.261
**Model fit**				
**Adjusted R²**	0.254		0.290	
**R²**	0.340		0.395	
**ΔR²**	—		0.055	
**Model p value**	0.001		—	
**p for F change**	—		0.106	

Note. Values are presented as unstandardized regression coefficients (B) with 95% confidence intervals (CI). This sensitivity analysis was conducted to assess whether micronutrient parameters explain the association between newly diagnosed CeD status and MoCA scores. Model 1 included age, income, physical activity, ferritin, folate, and vitamin B12 as covariates. Model 2 additionally included group status, with the control group as the reference category. Income reference category: below the national minimum wage. Statistically significant values are shown in bold. * $p < 0.05$; ** $p < 0.01$; *** $p < 0.001$. ND-CeD, newly diagnosed celiac disease; GFD-CeD, celiac disease on a gluten-free diet; MoCA, Montreal Cognitive Assessment.

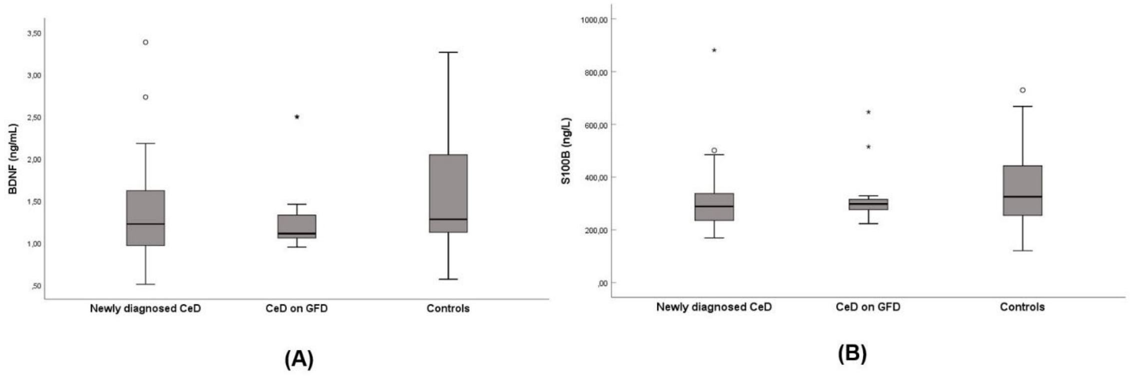

Supplementary Figure S1. Boxplots illustrating the distribution of serum BDNF and S100B levels across study groups (ND-CeD, n = 18; GFD-CeD, n = 17; controls, n = 27). No statistically significant between-group differences were observed (Kruskal-Wallis test: BDNF, H = 1.665, p = 0.435; S100B, H = 1.535, p = 0.464). Given the limited statistical power of the present study (sensitivity analysis: Cohen's f = 0.404), these null findings should not be interpreted as evidence of no difference.

BDNF, brain-derived neurotrophic factor; ND-CeD, newly diagnosed celiac disease; GFD-CeD, celiac disease on gluten-free diet.

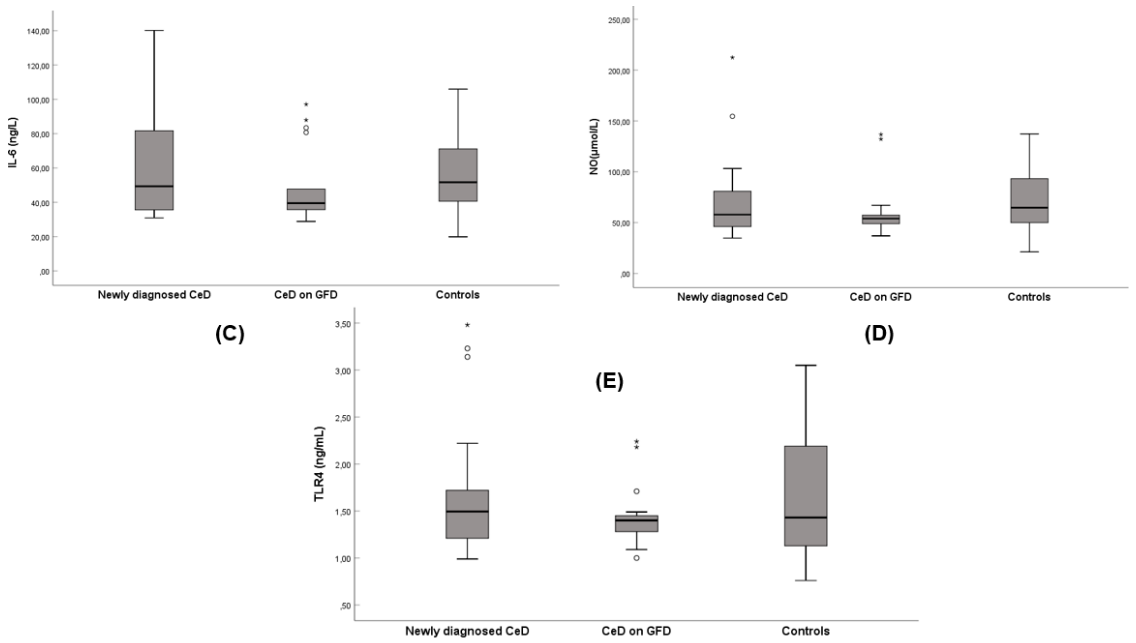

Supplementary Figure S2. Boxplots illustrating the distribution of serum IL-6, NO, and TLR-4 levels across study groups (ND-CeD, n = 18; GFD-CeD, n = 17; controls, n = 27). No statistically significant between-group differences were observed (Kruskal-Wallis test: IL-6, H = 1.868, p = 0.393; NO, H = 1.726, p = 0.422; TLR-4, H = 0.822, p = 0.663). Given the limited statistical power of the present study (sensitivity analysis: Cohen's f = 0.404), these null findings should not be interpreted as evidence of no difference.

IL-6, interleukin-6; NO, nitric oxide; TLR-4, toll-like receptor 4; ND-CeD, newly diagnosed celiac disease; GFD-CeD, celiac disease on gluten-free diet.
